# Supplementary material for: Longitudinal Motor Functional Outcomes and Magnetic Resonance Imaging Patterns of Muscle Involvement in Upper Limbs in Duchenne Muscular Dystrophy
Source: Medicina (Kaunas). 2021 Nov 18;57(11):1267. doi: 10.3390/medicina57111267 (PMC8624281; doi:10.3390/medicina57111267)
Supplement: Supplementary file 1 [file medicina-57-01267-s001.zip › medicina-1415911-supplementary.pdf]

Figure S1. PUL 2.0 Proforma Supplementary File

Performance of the Upper Limb Module for DMD 2.0 (PUL for DMD)

Name of patient (Initials): **DOB:** **DOA:**

Dominant arm (used for all tests): ☐Right ☐Left

Elbow extension ROM full = 0°: **Right:** **Left:**
e.g. 10° contracture = -10°
Supination ROM: Right: ☐Full ☐¾ ☐½ ☐¼ **Left:** ☐Full ☐¾ ☐½ ☐¼

☐Ambulant ☐Non-ambulant **Steroids:** ☐Yes ☐No

Spinal surgery: ☐Yes ☐No **Date of surgery:**

Name of evaluator: **Evaluator signature**

Entry item A. – start with A to identify starting point for subsequent tests. Circle score for each item. DO NOT INCLUDE IN TOTAL SCORE

Item	Description	0	1	2	3	4	5	6
A.	**Entry item**	No useful function of hands.	Can use hands to hold pen or pick up a coin or drive a powered chair	Can raise 1 or 2 hands to mouth but cannot raise a cup with a 200g weight in it to mouth	Can raise plastic cup with 200g weight in it to mouth using 1 or 2 hands	Can raise both arms (to shoulder height with or without compensation) .i.e. elbow bent or in extension	Can raise both arms simultaneously above head only by flexing the elbow (shortening circumference of the movement /using accessory muscles)	Can abduct both arms simultaneously elbows in extension in a full circle until they touch above the head.

For item A: A score of 3, 4, 5, 6 on item A, start with item 1 – on this page
A score of, 1, 2 start with item 7 on page 2

High level shoulder Dimension

Item	Description	0	1	2	Score
1<br>Score from Entry item above	**Shoulder abduction both arms above head**<br>"Raise your arms above your head out to the side – try and keep straight elbows"	Unable	Can raise both arms simultaneously **above head** only by flexing the elbow - **with** compensation	Can abduct both arms simultaneously elbows in extension in a full circle until they touch above the head	
2	**Raise both arms to shoulder height (elbows at shoulder height)**<br>"Raise your arms to shoulder level"	Unable	Can raise both arms to shoulder height either one at a time or with elbows flexed (**with compensation**)	Can raise both elbows to shoulder height **without** compensation e.g. simultaneously with elbows straight	
3	**Shoulder flexion to shoulder height (no weights)**<br>"Reach out and touch my hand" –elbow to eye level	Unable	Able **with** compensation	Able **without** compensation	
4	**Shoulder flexion to shoulder height with 500g weight**<br>"Reach out and touch my hand" –elbow to eye level	Unable	Able to lift 500g weight **with compensation**	Able to lift 500g weight **without compensation**	

High level shoulder Dimension (continued)

Item	Description	0	1	2	Score
5	**Shoulder flexion above shoulder height with 500 g weight** Hand on lap – "give me the weight"	Unable	Able to lift 500g weight **with compensation**	Able to lift 500g weight **without compensation**	
6	**Shoulder flexion above shoulder with 1 kg weight** Hand on lap – "give me the weight"	Unable	Able to lift 1 kg weight **with compensation**	Able to lift 1 kg weight **without compensation**	

Mid level elbow Dimension

Do these tests on all individuals

Item	Description	0	1	2	Score
7	**Hand(s) to mouth** "Bring the cup to your mouth with one hand"	Unable	Able to bring 200g in cup with any compensation to mouth (can use more than one hand and / or bring head to hands)	Able to bring 200g in cup to mouth with one hand no elbow support (without compensation)	
8	**Hands to table from lap** "Bring both hands from lap to table"	Unable	Able to bring two hands completely (to wrist crease) to table but **NOT simultaneously or in one action**	Two hands completely on table simultaneously	
9	**Move weight on table 100g** "Move the weight from outside circle to centre circle"	Unable	Can move **100g** weight from outer to centre circle using compensation (slide forearm or elbow make contact with table)	Can lift **100g** weight from outer to centre circle without compensation	
10	**Move weight on table 500g** "Move the weight from outside circle to centre circle"	Unable	Can move **500g** weight from outer to centre circle using compensation (slide forearm or elbow make contact with table)	Can lift **500g** weight from outer to centre circle without compensation	
11	**Move weight on table 1kg** "Move the weight from outside circle to centre circle"	Unable	Can move **1kg** weight from outer to centre circle using compensation (slide forearm or elbow make contact with table)	Can lift **1kg** weight from outer to centre circle without compensation	
12	**Lift heavy can diagonally** "Lift can from this circle nearest your hand to this circle furthest away and across your body"	Unable	Can move heavy can from nearest circle across body with compensation (slide forearm or elbow make contact with table)	Can lift heavy can from nearest circle across body without compensation	

Mid level elbow Dimension (continued)

Item	Description	0	1	2	Score
13	**Stack of three cans** "Stack these two cans, one at a time on the middle can using one hand"	Unable to stack third can even with compensation	Able to stack third can with compensation	Able to stack third can without compensation	
14	**Stack of five cans** "Stack these two additional cans, one at a time on top of this can using one hand"	Unable to stack fifth can even with compensation	Able to stack fifth can with compensation	Able to stack fifth can without compensation	
15	**Remove lid from container** "Use your hands to open this container"	Unable	Opens completely		

Distal wrist and hand Dimension **Do these tests on all individuals**

		0	1	2	Score
16	**Tearing paper** "Tear the sheet of paper beginning from here"	Unable	Tears the sheet of paper folded in half from the folded edge	Tears the sheet of paper folded in 4,beginning from the folded edge	
17	**Tracing path** "Use your pencil to complete the path in one smooth movement"	Unable	Completes the path with compensation - needs to raise pencil from paper or pivot arm	Able to complete the path **without stops** or raising hand from paper	
18	**Push on light** "Push on the light with the fingers of one hand"	Unable	Able to turn the light on momentarily with fingers of one hand	Able to turn the light on permanently with fingers of one hand	
19	**Supination** "Pick up the light and turn your hand over"	Unable	Picks up the light but either turns hands over incompletely or uses compensation to turn it over	Picks up the light, and turns the hand over completely with no compensatory movements	
20	**Picking up coins** "Using one hand, Pick up 6 coins, one at a time"	Cannot pick up one coin	Can pick up **one** coin/ token	Can pick up six coins in one hand	
21	**Placing finger on number diagram** (precision not essential) "Using one finger to touch each number on the diagram"	Cannot raise the finger or slide it on the diagram	Able to place finger (slide or lift) between at least two squares	Able to place finger successively on the numbers of the diagram (with or without compensation)	
22	**Pick up 10g weight finger pinch** "Pick up this small weight like this (by body of weight)"	Unable	Able to grip and lift weight off surface		

Additional Material

Item 17: Tracing a path

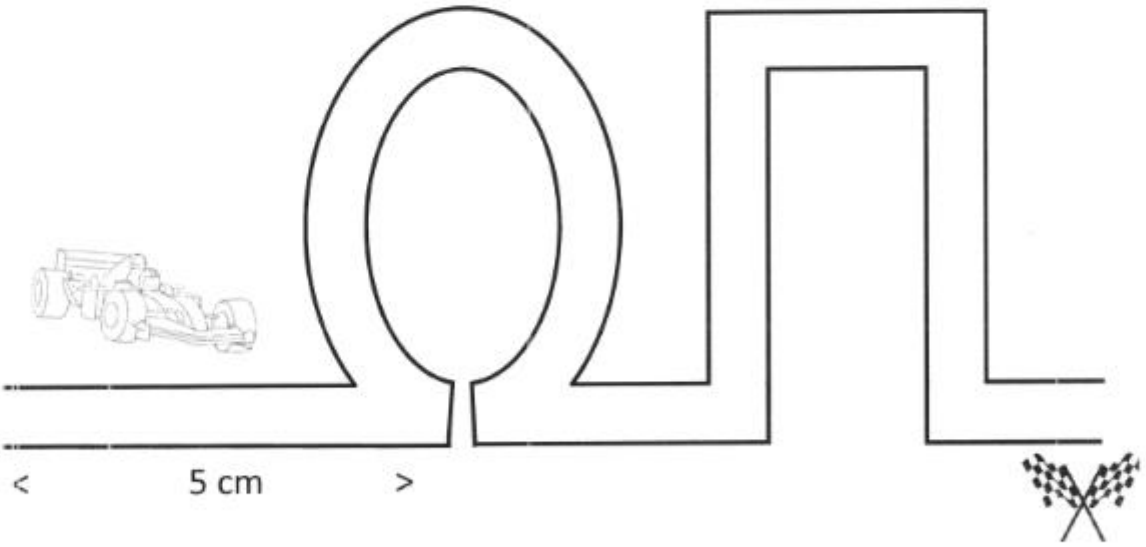

Item 21: Placing finger on number diagram

Instruction: Starting on the yellow number 1 point to the numbers 1 to 10 in turn following the arrow

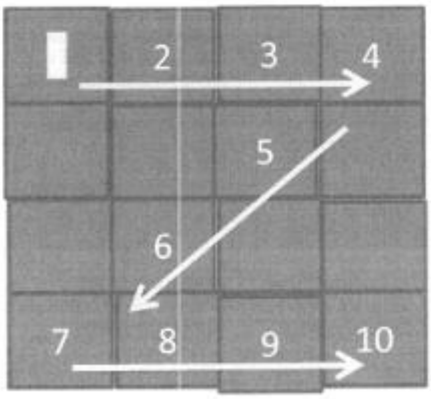

Figure S2. MRI and PUL scores at shoulder, arm and forearm level at baseline and at follow up

N	A/NA	AGE	SHOULDER MRI score at FOLLOW UP										ARM MRI score at FOLLOW UP			FOREARM MRI score at FOLLOW UP															PUL TOTAL SCORE			MRI TOTAL SCORE		
			del	suprasp	infrasp	subscap	pec minor	pec major	corac	serr	teres minor	lat	bic	brac	tri	sup	pron	F cr	Palm	F ds	F cu	F dp	Anc	E cu	E dm	Ed	E cr	Br R	F pl	E pl	BASELINE	FOLLOW UP	PUL changes	BASELINE	FOLLOW UP	MRI changes
1	A	5																													39	39	0	14.5	14.5	0
2	A	6																													41	42	1	12	12.5	0.5
3	A	8																													42	42	0	13	15	2
4	A	9																													40	36	-4	15	23	8
5	A	10																													42	42	0	15.5	18	2.5
6	A	10																													42	39	-3	53.5	56	2.5
7	A	10																													40	37	-3	20.5	29	8.5
8	EA	10.7																													36	31	-5	49.5	52	2.5
9	EA	11																													42	41	-1	36.5	38.5	2
10	A	11																													41	41	0	25	25	0
11	ENA	11.11																													27	26	-1	59.5	73.5	14
12	ENA	11.7																													37	28	-9	39	51	12
13	A	12																													42	42	0	17.5	18	0.5
14	ENA	12.5																													41	41	0	22.5	31.5	9
15	ENA	12.5																													29	25	-4	29	29	0
16	ENA	13																													35	31	-4	48	55.5	7.5
17	LNA	14.1																													36	31	-5	37.5	38.5	1
18	A	16																													41	41	0	18	18	0
19	LNA	16																													17	14	-3	87.5	87.5	0
20	ENA	17																													36	33	-3	56	57	1
21	LNA	18																													37	31	-6	64	64.5	0.5
22	LNA	18																													8	6	-2	110	110	0
23	LNA	20																													24	26	2	74.5	80.5	6
24	LNA	20																													25	22	-3	44	52	8
25	LNA	20																													15	13	-2	99	100	1
26	LNA	22.5																													13	12	-1	87.5	95.5	8
27	LNA	30																													16	14	-2	93	100.5	7.5
